# Improving read alignment through the generation of alternative reference via iterative strategy

**DOI:** 10.1038/s41598-020-74526-7

**Published:** 2020-10-30

**Authors:** Lina Bu, Qi Wang, Wenjin Gu, Ruifei Yang, Di Zhu, Zhuo Song, Xiaojun Liu, Yiqiang Zhao

**Affiliations:** 1 State Key Laboratory of Agrobiotechnology, College of Biological Sciences, China Agricultural University, Beijing, 100193 China; 2 Genetalks Biotech. Co., Ltd, Changsha, 410000 Hunan China; 3 College of Animal Science and Veterinary Medicine, Henan Agricultural University, Zhengzhou, 450000 Henan China

**Keywords:** Biological techniques, Bioinformatics

## Abstract

There is generally one standard reference sequence for each species. When extensive variations exist in other breeds of the species, it can lead to ambiguous alignment and inaccurate variant calling and, in turn, compromise the accuracy of downstream analysis. Here, with the help of the FPGA hardware platform, we present a method that generates an alternative reference via an iterative strategy to improve the read alignment for breeds that are genetically distant to the reference breed. Compared to the published reference genomes, by using the alternative reference sequences we built, the mapping rates of Chinese indigenous pigs and chickens were improved by 0.61–1.68% and 0.09–0.45%, respectively. These sequences also enable researchers to recover highly variable regions that could be missed using public reference sequences. We also determined that the optimal number of iterations needed to generate alternative reference sequences were seven and five for pigs and chickens, respectively. Our results show that, for genetically distant breeds, generating an alternative reference sequence can facilitate read alignment and variant calling and improve the accuracy of downstream analyses.

## Introduction

Whole-genome sequencing provides a comprehensive method to identify genomic variations^1,2^. As next-generation sequencing (NGS) has generated an ever-increasing volume of genomic data, it is not uncommon for current studies to involve hundreds or even thousands of individuals^3–5^. The accumulation of sequencing data provides great insight into biological problems and also improves the rigorousness and comprehensiveness of genomic analyses, especially for population genetics and association studies^6,7^. However, massive sequencing data processing and genome variant calling impose a heavy computational and storage burden and have thus become an emergent issue for large-scale studies. To eliminate computing bottlenecks, efforts have been made to develop more efficient algorithms or to utilize parallel computing^8^. Recently, heterogeneous computing with FPGA (Field-Programmable Gate Array) accelerators has shown significant potential to produce significant improvements in the computing efficiency of short-read alignment and variant calling while maintaining a very high level of consistency between the output and the original method^9–11^. For example, Menges F et al. proposed a new base-calling algorithm that was implemented in FPGA to achieve real-time performance^12^. Using FPGA, Arram J et al. accelerated the alignment of short reads 28 times faster than Bowtie2 running with 16 threads on dual Intel Xeon E5-2640 CPUs^13^. Acceleration via FPGA enables us to perform time consuming analyses that could not be done previously.

In addition to increasing computing efficiency, it is even more important to ensure that effective and accurate information is extracted from the sequencing data. Despite substantial genetic variations being found across breeds, only one complete reference genome is generally available for each species. For example, the reference genome for pigs came from a domesticated duroc pig^14,15^, while the reference genome for chickens is based on a wild red junglefowl^16,17^, which is the ancestor of the domestic chicken^18,19^. In most cases, the closest reference genome will be used for the read alignment and subsequent variant calling^20,21^. Unfortunately, in some cases, the closest reference genome is not very similar—especially for domesticated plants and animals, among which strong artificial and natural selection has led to extensive genetic differences between the domesticates and their wild counterparts or among different domesticated breeds. For example, a recent study on Chinese indigenous pigs identified more new SNPs (Single nucleotide polymorphisms) than those recorded in the dbSNP database^22^.

A high-quality and representative genome assembly can greatly facilitate studies. Currently, genome analyses at the population level often include individuals from multiple breeds. A genetic distance between the reference genome and the individual under investigation that is too large can lead to ambiguous alignment and inaccurate variant calling and, in turn, compromise the accuracy of the analysis. Although the costs of genome sequencing continue to fall, it remains unrealistic to build a reference genome for every breed of species since De Novo genome assembling is still technically cumbersome and very expensive. The generation of alternative reference sequences, therefore, is a cost-effective approach to satisfy the needs of this type of research. Cho et al. built a Korean consensus reference by incorporating common variants in the Korean population and found that a consensus reference can be beneficial for efficient variant detection^23^. By merging the alignment results, Okumur K et al. constructed alternative consensus references for Mycobacterium tuberculosis. An empirical evaluation showed that the use of a consensus reference significantly improved mapping efficacy and facilitated phylogenetic analysis^24^. Although researchers have realized that the standard reference genome might not always perform well for specific studies, there is still a lack of systematic research on which cases require an alternative reference sequence and how an alternative reference sequence should be generated.

In this study, with the help of the FPGA hardware platform, we performed an extensive evaluation of the generation of alternative reference sequences. Instead of using a one round substitution approach, we employed an iterative strategy, through which highly variable regions were recovered as the number of iterations increased. We showed that this process improves read alignment and variant calling not only for a genetically distant target breed but also for other breeds that are distant to the reference breed but close to the target breed. By considering a balance between sensitivity and computing costs, we also evaluated the optimal sequencing coverage and iterations that were required to generate an alternative reference sequence. Our results provide the first comprehensive study on the effective generation of an alternative reference sequence.

## Results

### Accurate variant calling by GTX-One

Since NA12878 is the gold standard publicly available variant set for variant caller benchmarking, to evaluate the performance of variant calling using the GTX-One platform, 30× (or 90 Gb) of whole genome sequencing data of NA12878 (H1) was used for variant calling using both the GTX-One and the GATK Best Practice (GPB) workflow. Based on the Genome in a Bottle Consortium (GIAB) gold standard callset, we defined the true positives (variants called with the same genotype as the gold standard callset, TP), false positives (variants called but not in the gold standard callset, FP), and false negatives (variants in the gold standard callset but not called, FN). We calculated the precision and sensitivity for both the GTX-One and GBP workflow using the following formulas: precision = TP/(TP + FP) and sensitivity = TP/(TP + FN). For SNPs, the GTX-One achieves a high precision of 99.54% and a high sensitivity of 99.36%. Overall, the performance of GTX-One is nearly identical to that of the GBP workflow, but it is much more efficient (Table 1 and Supplementary Table S1).

**Table 1:** A comparison of the FPGA and CPU implementation of variant calling of the NA12878 data.

Method	Variant type	FP	FN	TP	Precision	Sensitivity	Time (min)	Memory (GB)
GTX-One	InDel	8711	15,999	464,578	0.9816	0.9667	33	33.2
	SNP	14,706	20,601	3,188,714	0.9954	0.9936		
GBP	InDel	6339	11,312	469,265	0.9867	0.9765	1904	30
	SNP	9312	31,760	3,177,555	0.9971	0.9901		

### Differences in the mapping rates among domestic breeds

Domesticated plants and animals are subject to directional selection. Moreover, the demographic effects of isolation and genetic drift also change the allele frequencies of a population. Over time, these factors work together to promote genetic divergence across breeds. This is why the standard reference genome sequences do not always perform well. To reveal the differences in the mapping efficiency for distinct breeds, we chose genetically different Chinese domestic pigs and European commercial pigs, as well as Chinese domestic chickens and commercial layer chickens, for comparison. Since the quality of the reference genome can also affect mapping efficiency, we repeated the mapping process for both the latest and the previous versions of the genome assemblies.

For the pig species, we included five breeds and two reference genome versions: Sscrofa10.2 and Sscrofa11.1. Duroc is the breed from which the pig reference genome was built. Similarly, we included six breeds and two reference genome versions, galGal5 and galGal6, for chickens. Red junglefowl is the breed from which the chicken reference genome was built. As listed in Table 2 and Supplementary Table S2, even when controlled for the version of the reference genome, the mapping rates still varied among breeds of the same species. For pig species, the mapping rates of different breeds showed larger variations compared to those of chickens. As expected, for the commercial breeds DU and LD, the mapping rates were higher than those of Chinese indigenous breeds. For chicken species, the overall mapping rates were relatively higher and less variable among different breeds; however, approximate 2% differences were still observed between the best and worst scenarios. In addition, the results show that the mapping rate was significantly influenced by the quality of the reference assembly (see Supplementary Table S3 for statistics on the reference assemblies), indicating the necessity to use high-quality reference sequences for genomic analysis.

**Table 2:** Mapping rates for different pig breeds.

Reference	WZS (%)	BMX (%)	SZL (%)	LD (%)	DU (%)
Sscrofa10.2	87.61	87.56	88.31	90.08	90.02
Sscrofa11.1	94.74	93.82	95.78	96.75	97.16

We next downloaded the genome sequencing data for one human (H2) and one chimpanzee (C1) as a control to evaluate the degree of mapping differences. The genome sequencing data for the human and the chimpanzee were both purposely aligned to the GRCh37 and GRCh38 reference genome. Surprisingly, we found that the mapping rate of the chimpanzee data to the human genome was as high as 97.80%, which is only 2% less than human data, as listed in Table 3. The difference in the mapping rate between humans and chimpanzees is even smaller than the within-species differences we observed in the pig species, indicating that the genomes of domestic animals changed significantly during the process of domestication. Thus, if only the standard reference genome sequences were used, the analysis results might be compromised, especially for studies including multiple domesticated breeds.

**Table 3:** Mapping rates for chimpanzee and human.

Reference	Human (%)	Chimpanzee (%)
GRCh37	99.69	97.64
GRCh38	99.79	97.80

### Optimal sequencing coverage for accurate genotype calls

At higher levels of genome coverage, the called variants afford a higher degree of confidence because each base is covered by a greater number of aligned reads. However, a higher coverage of sequencing means higher costs, while sequencing coverage that is too low often causes inaccurate genotype calls. Based on the above results, high-quality reference genome assemblies, Sscrofa11.1 and galGal6, were used for further analyses of pigs and chickens, respectively. Using WZS as an example, the statistics in Table 4 show that with an increase in sequencing coverage, both the number of variants called, and the sensitivity increased. The mapping rate, however, remained consistently high for all sequencing coverage rates, suggesting good and stable mapping quality. Statistics for the other pig and chicken breeds are listed in Supplementary Table S4 and Supplementary Table S5.

**Table 4:** Summary of the mapping statistics for WZS at different sequencing coverages.

WZS					
Coverage (SNP)	Number of SNP/small InDel	Sensitivity (SNP) (%)	Sensitivity (InDel) (%)	Mapping rate (%)	Coverage ratio (%)
1	1,944,247	13.99	9.34	94.48	53.76
2	4,712,874	34.49	24.91	94.48	76.63
3	6,889,545	51.24	38.88	94.48	86.84
4	8,395,125	63.55	50.06	94.48	91.66
5	9,379,364	71.21	57.80	94.49	94.07
6	10,055,111	75.99	64.26	94.49	95.35
7	10,493,413	80.99	68.29	94.49	96.08
8	10,785,210	83.11	71.00	94.49	96.53
9	11,057,014	84.46	74.60	94.48	96.82
10	11,212,461	87.27	76.15	94.48	97.02
11	11,384,781	87.90	78.79	94.48	97.16
12	11,475,145	88.32	79.71	94.48	97.27
13	11,539,920	90.31	80.38	94.48	97.36
14	11,653,799	90.50	82.42	94.48	97.43
15	11,694,650	90.62	82.84	94.48	97.48
16	11,782,668	92.13	84.52	94.48	97.53
17	11,805,726	92.19	84.77	94.48	97.57
18	11,827,889	92.18	84.99	94.49	97.60
19	11,898,117	92.15	86.41	94.49	97.63
20	11,908,403	93.37	86.52	94.48	97.66
21	11,970,269	93.34	87.74	94.48	97.68
22	11,974,123	93.26	87.79	94.49	97.70
23	11,978,131	94.28	87.83	94.48	97.72
24	12,029,145	94.21	88.95	94.49	97.74
25	12,030,019	94.12	88.91	94.48	97.75
26	12,075,970	94.99	89.88	94.48	97.77
27	12,073,458	94.92	89.86	94.48	97.78
28	12,071,291	94.80	89.81	94.49	97.79
29	12,113,790	94.72	90.71	94.49	97.80
30	12,108,531	95.50	90.64	94.48	97.82

**Figure 1:**
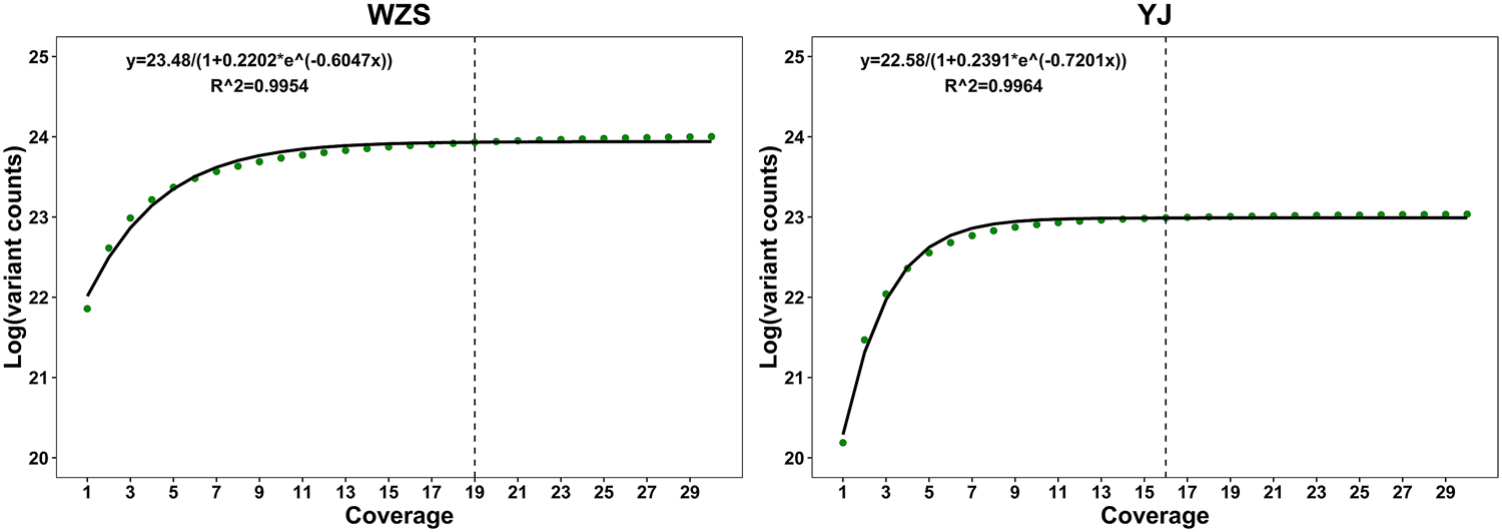
Variant counts against the sequencing coverage of WZS and YJ. The dashed line indicates the optimal sequencing coverage. The x-axis is the coverage (SNP), and the y-axis is the logarithm of the variant counts. The equation in the figure is the fitted equation. The green dots represent true variant counts, and the black curve is the fitted curve.

To determine the optimal genome sequencing coverage for accurate genotype calls, we plotted the variation counts (log-transformed) against the coverage. The curve was found to be best fitted by a logistic function (Fig. 1, Supplementary Fig. S1 and Supplementary Fig. S2). We thus determined the threshold to be 0.0001 by selecting the slope of the tangent of the curve, which corresponds to 18.2× (rounded up to 19×) in pigs (Table 5) and to 15.4× (rounded up to 16×) in chickens (Supplementary Table S6). We applied the same method to the sensitivity and coverage ratio, and the results were similar. Thus, we report the results using variation counts unless otherwise specified.

**Table 5:** Function of the fitted curve and the optimal sequencing coverage in pig breeds.

Breeds	Function	R^2^	Slope of the tangent		
			17×	18.2×	19×
WZS	$\frac{23.48}{1+0.2202*{e}^{-0.6047x}}$	0.9954	0.0001	0.00005	0.00003
BMX	$\frac{23.23}{1+0.2204*{e}^{-0.6587x}}$	0.9947	0.00005	0.00002	0.00001
SZL	$\frac{23.25}{1+0.2057*{e}^{-0.6595x}}$	0.9953	0.00004	0.00002	0.00001
LD	$\frac{22.54}{1+0.2362*{e}^{-0.6831x}}$	0.9965	0.00003	0.00001	0.000006
DU	$\frac{22.02}{1+0.2063*{e}^{-0.5551x}}$	0.9479	0.0002	0.0001	0.00007

### Improving mapping by using alternative references generated by an iterative strategy

**Figure 2:**
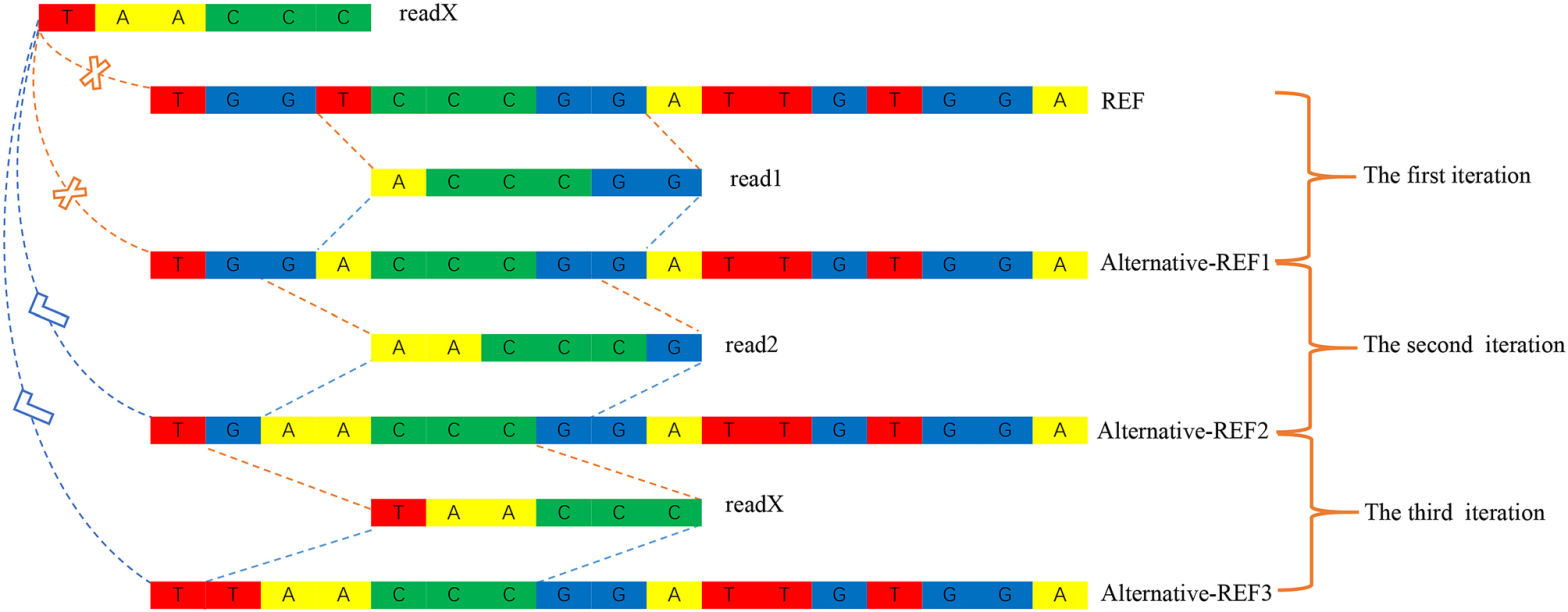
Iterative substitutions of the reference sequence enable the mapping of readX, which could not be mapped before due to consecutive mismatches**.** In the case of allowing one base mismatch, in the first iteration, read1 was mapped to the original reference genome REF, and the genome Alternative-REF1 was generated by base replacement; in the second iteration, read2 was mapped to Alternative-REF1, and the genome Alternative-REF2 was generated by base replacement; after two iterations, readX was mapped to Alternative-REF2, and the genome Alternative-REF3 was generated by base replacement. REF indicates the reference genome, and Alternative-REF1/Alternative-REF2/Alternative-REF3 indicates the alternative reference sequence in different iterative rounds.

In short, the aim of generating alternative references is to increase the mapping rate. For highly divergent regions containing consecutive mismatches, the sequencing reads are not directly mappable, so substitutions to the reference base cannot be easily made. To solve this problem, we employed an iterative strategy for which the consecutive mismatches are substituted step by step, as illustrated in Fig. 2.

After the optimal sequencing coverage was determined, the raw reads were sampled at the optimal coverage for variant calling. The callset was then used as the input for the GATK FastaAlternateReferenceMaker function to generate a new reference sequence. In the first round of iterations, the publicly released reference genome sequence was used. In the following iterations, the updated reference and the callset from the updated reference were used as inputs for the next iteration. Since we had no prior knowledge of how many iterations would be enough, we performed 30 iterations for both species. To test how an alternative reference sequence improves mapping, in each round, the updated WZS alternative reference sequence was recorded, and the sequencing reads from BMX, SZL, and LD pig breeds were mapped against it. The same was done for the updated YJ alternative reference sequence for chickens, and the sequencing reads from the LS, LDH, and BLK chicken breeds were mapped against it. As shown in Table 6 and Supplementary Table S7, increased mapping rates were observed for the alternative references. The maximum increases in the mapping rate for pigs were 1.51% for WZS, a 1.16% increase for BMX, and 1.21% for SZL. For chicken breeds, the maximum increases in the mapping rate were 0.18% for YJ, 0.42% for ZJ, and 0.37% for LS. Statistics of the variant counts, mapping rates, and coverage ratios for the other breeds are detailed in Supplementary Tables S7 and S8. To exclude the possibility that the increased mapping rate is caused by chimeric sequences, we checked if the alternative reference sequence produced actually would become closer to the target breed. We aligned the progressive WZS alternative reference sequences against the public WGZ genome using Minimap2. As shown in table S9, the alternative reference genome became progressively more similar to the WZS genome. Overall, using alternative reference sequences improved mapping for genetically distant breeds, which would help researchers analyze population level data more effectively.

**Table 6:** Summary of the mapping statistics for WZS and BMX.

Number of iterations	WZS^0^			WZS^1^			BMX^1^		
	Variant counts	Mapping rate (%)	Coverage ratio (%)	Variant counts	Mapping rate (%)	Coverage ratio (%)	Variant counts	Mapping rate (%)	Coverage ratio (%)
1	14,311,513	94.48	97.63	14,858,156	95.28	97.81	15,635,623	93.81	97.63
2	3,295,192	95.44	97.61	7,243,054	96.01	97.81	10,291,091	94.35	97.64
3	2,766,075	95.67	97.61	6,939,344	96.24	97.81	9,709,396	94.54	97.64
4	2,682,333	95.74	97.61	7,018,824	96.31	97.81	9,994,312	94.55	97.64
5	2,663,703	95.84	97.61	6,890,396	96.41	97.81	9,681,308	94.66	97.64
6	2,654,321	95.89	97.61	7,000,062	96.48	97.81	9,981,678	94.68	97.64
7	2,648,887	95.96	97.61	6,882,470	96.55	97.81	9,676,056	94.77	97.64
8	2,646,651	95.98	97.61	6,994,959	96.57	97.81	9,979,414	94.77	97.64
9	2,644,442	96.00	97.61	6,879,559	96.60	97.81	9,675,444	94.81	97.64
10	2,643,524	96.01	97.61	6,993,579	96.59	97.81	9,978,295	94.78	97.64
11	2,643,077	96.02	97.61	6,878,235	96.61	97.81	9,674,677	94.81	97.64
12	2,642,597	96.04	97.61	6,993,224	96.62	97.81	9,978,507	94.80	97.64
13	2,642,070	96.06	97.61	6,878,417	96.66	97.81	9,675,291	94.86	97.64
14	2,642,098	96.07	97.61	6,992,264	96.67	97.81	9,978,536	94.85	97.64
15	2,642,033	96.07	97.61	6,878,487	96.68	97.81	9,675,171	94.88	97.64
16	2,641,897	96.08	97.61	6,992,676	96.69	97.81	9,978,915	94.86	97.64
17	2,641,860	96.09	97.61	6,878,502	96.70	97.81	9,675,531	94.89	97.64
18	2,641,655	96.09	97.61	6,992,575	96.69	97.81	9,978,580	94.87	97.64
19	2,641,151	96.10	97.61	6,878,334	96.70	97.81	9,675,100	94.90	97.64
20	2,641,564	96.10	97.61	6,992,421	96.70	97.81	9,979,088	94.87	97.64
21	2,641,376	96.12	97.61	6,878,399	96.73	97.81	9,675,677	94.92	97.64
22	2,641,402	96.10	97.61	6,992,134	96.69	97.81	9,978,451	94.87	97.64
23	2,641,172	96.12	97.61	6,877,922	96.73	97.81	9,675,169	94.92	97.64
24	2,641,227	96.11	97.61	6,992,524	96.71	97.81	9,978,918	94.88	97.64
25	2,641,079	96.13	97.61	6,878,267	96.73	97.81	9,675,566	94.92	97.64
26	2,641,212	96.11	97.61	6,992,168	96.72	97.81	9,978,784	94.88	97.64
27	2,640,874	96.14	97.61	6,878,056	96.77	97.81	9,674,875	94.95	97.64
28	2,640,890	96.14	97.61	6,992,191	96.77	97.81	9,978,828	94.92	97.64
29	2,641,280	96.16	97.61	6,878,291	96.79	97.81	9,675,597	94.97	97.64
30	2,641,270	96.14	97.63	6,992,372	96.77	97.81	9,979,020	94.93	97.64

^0^Indicates the individual used for generating the alternative-reference sequence; ^1^indicates unrelated individual for reads alignment only to the alternative-reference sequence.

We noticed that the variant counts of WZS and BMX in Table 6 moved up and down during the iterations. Thus, we manually checked the output VCF files during each iteration. For the examples shown in Supplementary Table S10, we found that inconsistencies were caused by the switching over of heterozygous alleles in the individuals used to generate the alternative reference sequences, while the same loci in unrelated individuals, for read alignment, were only homozygous. This suggests that substitution to the reference base would only be meaningful for homozygous loci.

**Figure 3:**
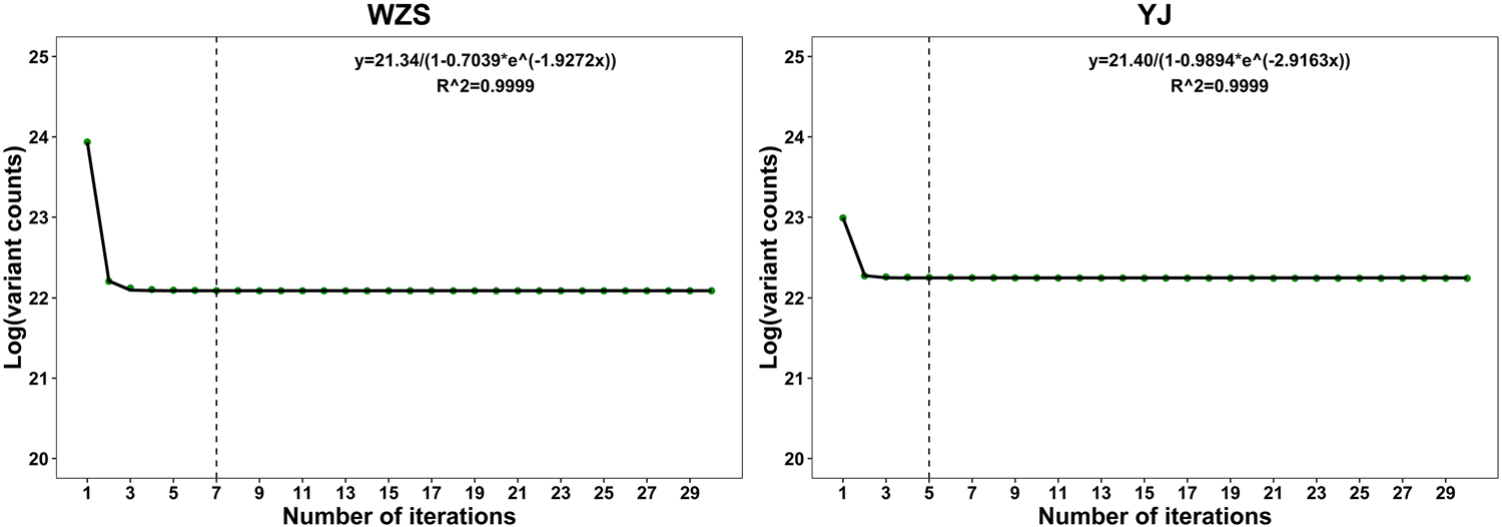
Variation counts against the number of iterations. The dashed line indicates the optimal iterations. The x-axis is the number of iterations, and the y-axis is a logarithm of the variant counts. The equation in the figure is the fitted equation, the green dots represent the true variant counts, and the black curve is the fitted curve.

Since the iteration process is time consuming and the drops in variation counts with additional iterations (WZS^0^ in Table 6) become less significant, to determine the optimal iteration number, we fitted the variation counts against the number of iterations. Again, the best fitting model was a logistic function (Fig. 3). Since the tangent to the curve approaches infinitely close to zero, we determined a threshold of -0.0001, which corresponds to seven iterations for pigs and five iterations for chickens (Table 7 and Supplementary Table S11).

**Table 7:** Function of the fitted curve and optimal number of iterations for WZS.

Breed	Function	R^2^	Slope of the tangent			
			4th round	5th round	6th round	7th round
WZS	$\frac{21.34}{1-0.7039*{\mathrm{e}}^{-1.9272x}}$	0.9999	− 0.013	− 0.002	− 0.0003	− 0.00004

**Figure 4:**
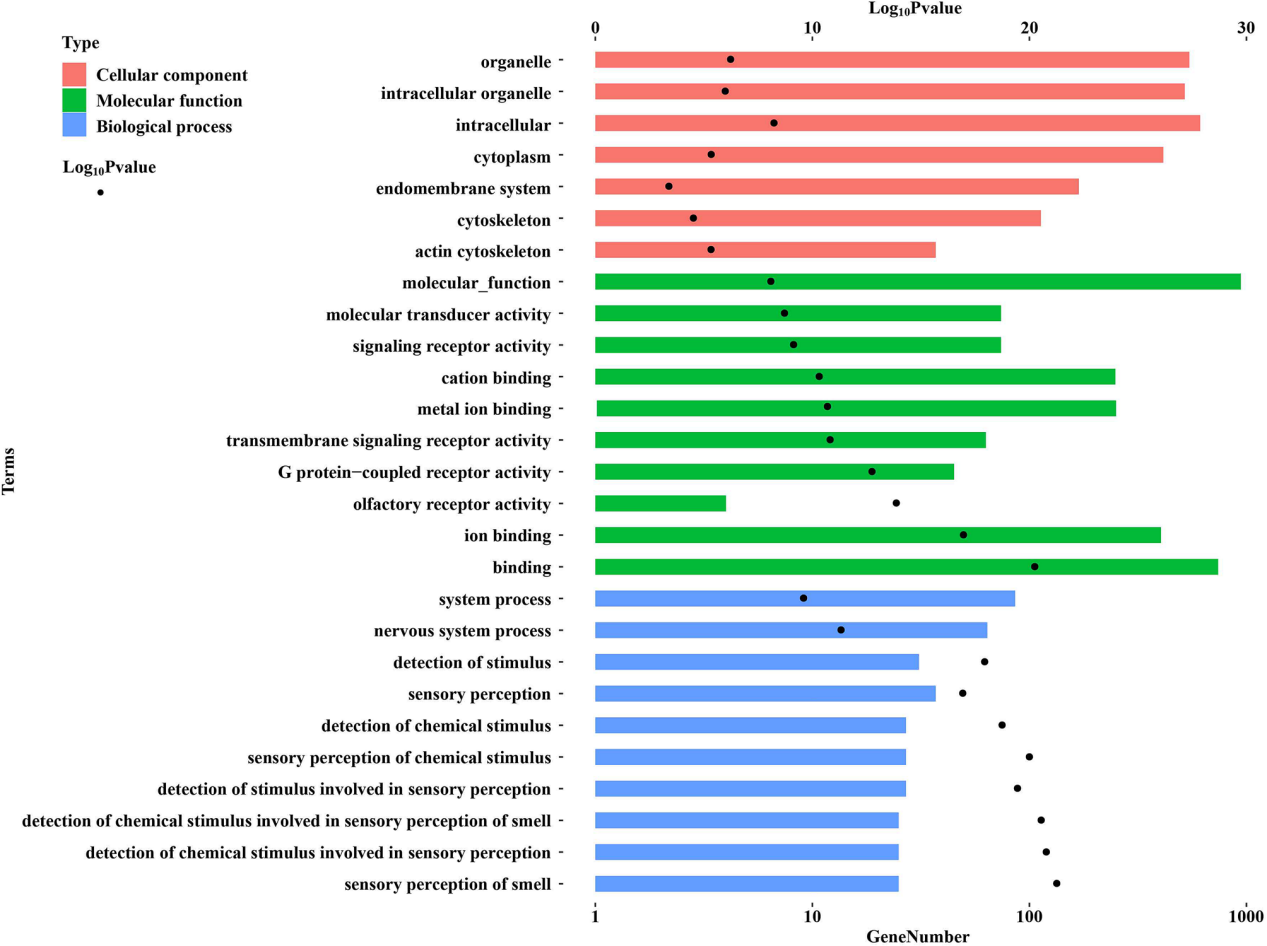
The result of the GO enrichment analysis.

For both BWA-MEM and GTX, we confirmed increased mapping rate upon the final WZS alternative reference sequences, using sequencing data of an unrelated WZS sample (Supplementary Table S12). We also discovered newly mapped sequences and novel high-quality variants that can only be identified using our alternative reference sequence (Supplementary Table S13 and S14). By aligning the WZS alternative reference sequence with the original pig reference sequence, we identified 138,545 highly variable regions (HVR) recovered by our iterative strategy, among which 44.76% were overlapped with genes, and 33.59% were overlapped with CDSs (detailed in Table 8). The GO (Gene Ontology) analysis results showed that the genes overlapping with HVR were mostly enriched in sensory perception (Fig. 4), which is consistent with previous reports that the genes related to sensory perception experience rapid evolution^25^.

**Table 8:** Highly variable regions between the alternative-reference sequence and the original reference sequence.

Chr	Number of HVR	Number of HVR overlapped with gene	Number of HVR overlapped with CDS
chr1	8551	1324	1114
chr2	16,963	9541	6030
chr3	13,377	8571	6217
chr4	15,088	8879	16,147
chr5	2699	1175	883
chr6	10,674	2538	1393
chr7	17,697	7742	6409
chr8	4346	1467	206
chr9	3785	1401	269
chr10	2773	1218	509
chr11	2182	1140	222
chr12	9785	6635	1812
chr13	1816	740	203
chr14	12,666	3692	2867
chr15	3432	485	81
chr16	1023	219	38
chr17	2730	134	13
chr18	8958	5105	2121

## Discussion

Due to the rapid development of sequencing technologies, the volume of sequencing increases rapidly, while the price per base pair continues to fall. However, it is always important to sequence with optimal coverage to reach a balance between the cost and accuracy of the analysis. For low-coverage sequencing, some regions of the genome might not be covered, or heterozygote sites might be misgenotyped as homozygous^26^. Ultra-high coverage sequencing will, on the other hand, greatly increase the costs for both sequencing and the data analysis. Here, the optimal sequencing coverage of accurate genotype calls for pigs and chickens was 19× and 16×, respectively. This will provide a useful reference for related studies.

However, obtaining accurate variant information not only depends on sequencing coverage but also on good reference genomes. Since the standard reference genome might not satisfy the special needs of different studies, in some studies, researchers have attempted to look for alternative reference genomes. For example, Ai H et al. studied the domestication of Chinese pigs. In their work, the genome sequences for WZS, instead of the published Duroc assembly, were used as the reference to ensure a better alignment with Chinese pigs^22^. Incarnato et al. created an alternative reference sequence for the E14 genome based on the mm9 assembly. The sequencing reads mapped to the E14 genome increased by around 5% compared to those of the mouse mm9 genome^27^. In this study, by using alternative reference sequences, the mapping rate increased by 0.61–1.68% for Chinese pig breeds and by 0.09–0.45% for Chinese chicken breeds. Compared to the chickens that were domesticated from red junglefowl in southeast Asia, the domestication of the pig took place independently in two locations: East Anatolia and China^28^. Our result thus suggests that the generation of alternative reference sequences is more necessary for species with complex genetic backgrounds. Unlike previous methods that used single step substitution, we employed an iterative strategy of generation to produce alternative reference sequences. Although here the GTX-one performs the sequence mapping and variant calling processes, we confirmed that this iterative strategy can cooperate with other mappers/callers (Supplementary Table S15). The alternative reference sequences generated by this approach enable the substitutions of consecutive mismatches in highly variable regions. These regions, where sequencing reads are not directly mappable, could cause a complete loss of information and leave a false impression that the region is highly conserved.

We found that several highly variable regions overlap with the genes or even CDSs, indicating a possible overestimation of the conversation of coding sequences during domestication. Our method corrected this problem and recovered phylogenetically informative sites that are missed by using public reference sequences, which could improve the accuracy of downstream analyses, including GWAS and genetic diversity evaluations. The GO enrichment analysis of the genes in highly variable regions were found to be enriched in sensory perception pathways, suggesting that, even though previous studies reported that the genes involved in sensory perception are among the most rapidly evolving genes^25^, this phenomenon might still be underestimated.

Our results indicate that alternative reference sequences are not only effective for improving the mapping rates of the breeds used to generate the alternative reference sequences but are also effective for other genetically close populations. Interestingly, we found that the variation count reported in the second round of iterations was significantly lower than that obtained during the first round, indicating a substantial number of fixed substitutions across distinct breeds. These fixed substitutions are not informative for either the target breed or other genetically close breeds and can be avoided when using an alternative reference sequence.

In summary, our iterative strategy for generating alternative reference sequences facilities the read alignment for genetically distant breeds and will improve all variant-based downstream analyses, especially for population genetics analyses.

## Materials and methods

### Samples

The whole genome sequencing data for 6 pigs, 7 chickens, 2 humans, and 1 chimpanzee were used in this study. These data were either downloaded from the NCBI SRA database (https://www.ncbi.nlm.nih.gov/) and DDBJ (https://ddbj.nig.ac.jp/DRASearch/) or sequenced by our lab. The details of the samples are shown in Table 9.

**Table 9:** Details of the samples used in this study.

Sample name	Abbreviation	Location	Sequencing coverage	Data Source
Wuzhishan	WZS^0^	Hainan (Pig)	76×	SRR448574 SRR448575 SRR448578 SRR448581 SRR448586 SRR448588 SRR448589 SRR448591
Wuzhishan	WZS^1^	Hainan (Pig)	35×	This study
Bamaxiang	BMX	Guangxi (Pig)	47×	This study
Shaziling	SZL	Hunan (Pig)	109×	This study
Duroc	DU	America (Pig)	36×	SRR8270382
Landrace	LD	Denmark (Pig)	103×	This study
Red junglefowl	RJF	Indonesia (Chicken)	12×	DRA003951
Zangji	ZJ	Tibet (Chicken)	122×	This study
Los island red	LDH	America (Chicken)	30×	This study
White plymouth rock	BLK	Europe (Chicken)	129×	This study
Beijing fatty	YJ^0^	Beijing (Chicken)	41×	This study
Beijing fatty	YJ^1^	Beijing (Chicken)	35×	This study
Langshan	LS	Jiangsu (Chicken)	120×	This study
Human	H1	Utah (Human)	30×	RMNISTHS_30xdownsample.bam
Human	H2	Canada (Human)	49×	SRR8595488
Chimpanzee	C1	Midwest Africa (Chimp)	41×	ERR2020658

### Read mapping and variant calling

GTX-One by the Genetalks company, a commercially available FPGA-based hardware accelerator platform, was used in this study for both the read alignment and variant calling. The alignment process for GTX-One is accelerated by the FPGA implementation of the parallel seed-and-extend approach based on the Smith–Waterman algorithm, while the variant calling process is accelerated by the FPGA implementation of the Genome Analysis Toolkit (GATK 3.7)^29^ HaplotypeCaller (Pair-HMM) (see Supplementary Text for details). Based on the Genome in a Bottle Consortium (GIAB) gold standard callset, we evaluated the performance of both the GTX and the BWA-GATK "Best Practices" workflow (GBP, BWA 0.7.17 + GATK 3.7) in terms of precision and sensitivity, using the following formulas: precision = TP/(TP + FP) and sensitivity = TP/(TP + FN), where TP represents true positive (variants called with the same genotype as the gold standard callset), FP represents false positive (variants called but not in the gold standard callset) and FN represents false negative (variants in the gold standard callset but not called).

### Determining the optimal genome sequencing coverage

Raw reads were randomly sampled at 1× to 30× genome coverage with an increment of 1×. For each coverage, the sequencing data were aligned back to the reference genome, and variations were called using GTX-One. Variant counts, mapping rates (defined as the ratio of mapped reads to the total reads), and coverage ratios (defined as the proportion of the loci at a coverage greater than or equal to 1 compared to the reference genome) were recorded. Variant sets reported by the dataset to have excessive coverage (40× or higher) were used as gold standard. The BCFtools isec function^30^ was used to compare the gold standard VCFs for each sequence’s coverage, and we reported the sensitivity using the formula sensitivity = TP/(TP + FN), where TP represents a true positive (variants called with the same genotype as the gold standard callset), and FN represents a false negative (variants in the gold standard callset but not called). CurveExpert1.4 (https://www.curveexpert.net) was used to fit the variant counts against the coverage. We determined the optimal genome sequencing coverage by selecting the tangent slope of the curve at a threshold of 0.0001 using an in-house python script.

### Iterative strategy for the generation of alternative reference sequences

Given a specific variant callset (in VCF format), the GATK FastaAlternateReferenceMaker was used to replace the reference bases with the variations recorded in the callset to generate a new reference sequence. For each round, the variant callset was generated by GTX-One against the previous reference sequence. The reference sequence was continually updated during the iterative process. We selected WZS and YJ as examples for pigs and chickens, respectively, and, for both species, the iterative process continued for 30 rounds. Variant counts, mapping rates, and coverage ratios were recorded. Similarly, CurveExpert1.4 was used to fit the variant counts against the number of iterations to determine the optimal number of iterations. After the optimal number of iterations was determined, the final alternative reference sequence for the target breed was reported.

### Whole genome alignments for identifying highly variable regions and genome similarities

LASTZ^31^ was used to align the final alternative reference sequences and original reference sequences chromosome by chromosome. By parsing the alignment output of LASTZ, we reported highly variable regions that contain three or more consecutive mismatches. The gene-based annotations for highly variable regions were produced using the coordinate information in the genomic GTF file from the Ensembl repository. Function-based annotations were then based on gene-based annotations using Gene ontologies via PANTHER (https://pantherdb.org/). We also used Minimap2^32^ to align the Duroc and the WZS alternative reference sequences from each iteration against the public WZS genome. The genomic similarities were calculated as the number of matched bases divided by the total genome size according to the Minimap2 output.

### Ethical approval

This study was approved by the Animal Welfare Committee of China Agricultural University. The approval number is SKLAB-2012-11. All pigs and chickens used in this study were taken care and operated on according to the relevant regulations.

## Supplementary information

Supplementary text and figures

Supplementary tables
