# Supplementary material for: Improving read alignment through the generation of alternative reference via iterative strategy
Source: Sci Rep. 2020 Oct 30;10:18712. doi: 10.1038/s41598-020-74526-7 (PMC7599232; doi:10.1038/s41598-020-74526-7)
Supplement: Supplementary file 1 — Supplementary text and figures [file 41598_2020_74526_MOESM1_ESM.pdf]

Improving read alignment through the generation of alternative reference via iterative strategy

Lina Bu[1], Qi Wang[1], Wenjin Gu[1], Ruifei Yang[1], Di Zhu[1], Zhuo Song[2], Xiaojun Liu[3], Yiqiang Zhao[1*]

[1]State Key Laboratory of Agrobiotechnology, College of Biological Sciences, China Agricultural University, Beijing, 100193, China

[2]Genetalks Biotech. Co., Ltd., Hunan, 410000, China

[3]College of Animal Science and Veterinary Medicine, Henan Agricultural University, Henan, 450000, China.

* yiqiangz@cau.edu.cn

Supplemental Information

Includes:

Supplemental Figures (Fig. S1-S2)

Supplemental Tables (Tables S1-S8)

Supplementary Text

GTX-One perform the whole mapping process by synergy execution of CPU and custom FPGA logic. The software layer reads sequences from FASTQ files and efficiently finds the corresponding sequences' chains concurrently. The dynamic extension of seeds base on Smith Waterman algorithm, which is the most compute-intensive part in mapping process, is accelerated by customized FPGA logic. For each batch of seeds, the CPU encodes the read and reference sequences and corresponding control information and transmits them to PCIe. The data transfer between the CPU and the FPGA accelerator uses DMA and double buffering. The DMA mechanism is used to reduce the transmission overhead, while the double buffering mechanism is used to hide the delay of calculation and transmission. A large number of computing units (PEs) are integrated inside the FPGA accelerator, and each 32 PEs form an acceleration array, and multiple acceleration arrays are concurrently executed to accelerate the extension of the seed. The accelerator's transmit slot mechanism allows the CPU to use the data stream to drive the FPGA accelerator to start multiple tasks at once. The accelerator automatically performs task-free scheduling on tasks, making full use of all computing units to improve task execution efficiency. After the FPGA SW accelerator completes the dynamic extension, the candidate mapping locations are returned to the host memory. The insert size is calculated and the optimal mapping is selected based on the insert size distribution and SW extension scores.

After mapping, the alignments are sorted by reference positions and PCR duplicates are flagged. All alignments are divided into genomic regions with size of 4M and cached to SSD, which provides the I/O bandwidth necessary to feed the processing pipeline. For the read pair across two regions, a cross-repetition cache strategy is adopted to eliminate the data dependency of the pair-end duplicates marking. The advantages are as follows: (1) each genomic region can be sorted and duplicates-marked in parallel to maximize the use of CPU resources; (2) sorting, duplicates marking and subsequent variants calling can be executed in a pipeline, and the calculation time is hidden to a maximum extent. During sorting and duplicates marking, QC metrics such as mapping rate, sequencing depth, sequencing coverage and PCR duplicates ratio are output. These statistical metrics are consistent with the results of widely-used tools such as SAMtools and Picard (http://broadinstitute.github.io/picard/).

The GTX-One variants caller fully implements the algorithm of GATK HaplotypeCaller. Firstly, it

defines the "active regions" of mutations on the genome. These active regions are likely to have potential mutations. Subsequent calculations only need to be performed for these regions. Secondly, for each active region, the reads are assembled to obtain a set of possible haplotypes. These haplotypes are aligned with the reference genome using the Smith Waterman algorithm to obtain potential mutations, and the original alignments between reads and reference are updated simultaneously. For each pair of read-haplotypes, the probability P(r|H) is calculated by the pairHMM algorithm, that is, the conditional probability of read is observed assuming that haplotype is the true haplotype. Finally, for each mutation position of the active region, the probabilities of all possible genotypes are calculated based on Bayesian formula and genotypes with the maximum probability is took as the final calling result. The whole calling process is driven by a highly-optimized software layer, while the most time consuming pairHMM calculations are accelerated by FPGA. The pairHMM software controller works similarly as the SW accelerator of reads mapping. GTX-One variants caller executes concurrently by genomic chromosomes in a multi-threaded manner. By designing a reasonable task scheduling strategy, the load imbalance caused by different chromosome sizes is eliminated. The final detected SNVs and short InDels are recorded to VCF file.

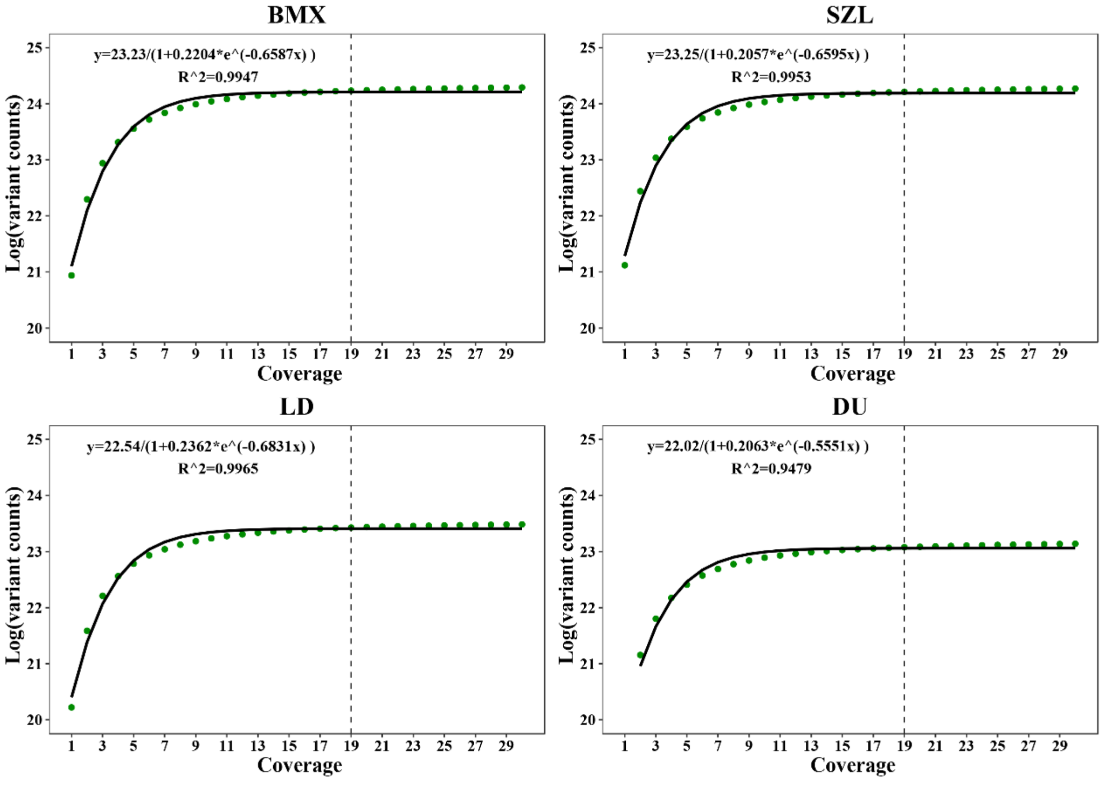

Supplementary Figure S1. Variant counts against sequencing coverage of pigs. The dash line

indicates the optimal sequencing coverage. The x-axis is the coverage (SNP), and the y-axis is the logarithm of the variant counts, the equation in the figure is the fitted equation, green dots represents true variant counts, the black curve is the fitted curve.

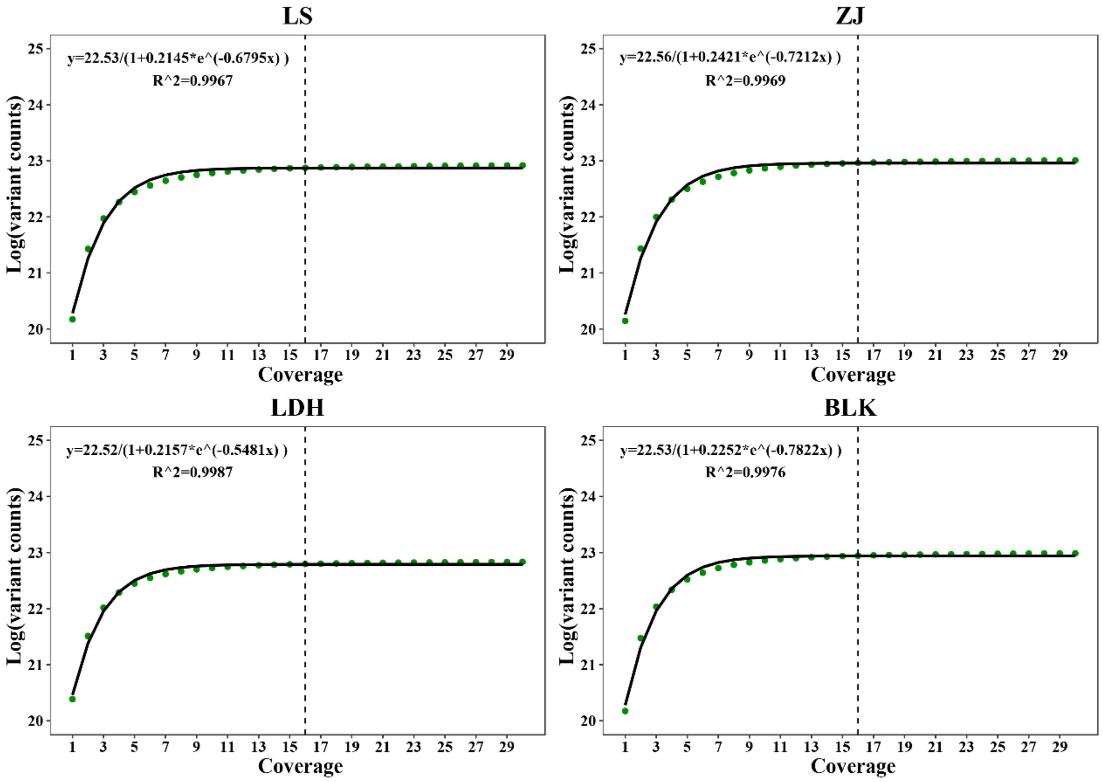

Supplementary Figure S2. Variant counts against sequencing coverage of and chickens. The dash line indicates the optimal sequencing coverage. The x-axis is the coverage (SNP), and the y-axis is the logarithm of the variant counts, the equation in the figure is the fitted equation, green dots represents true variant counts, the black curve is the fitted curve.
